# Case report: Cervical brachytherapy technique for locally advanced cervical cancer in a patient with complete bicorporeal uterus

**DOI:** 10.3389/fonc.2024.1361562

**Published:** 2024-06-05

**Authors:** Lipeng Ding, Zhangcai Zheng, Yueao Cao, Zhenjiang Wang, Baoyu Zhu, Guoying Miao, Wenzhen Yuan

**Affiliations:** ^1^ Department of Radiation Oncology, Gansu Provincial Maternity and Child-care Health Hospital, Lanzhou, China; ^2^ The First School of Clinical Medicine, Lanzhou University, Lanzhou, China; ^3^ The Department of Oncology, The First Hospital of Lanzhou University, Lanzhou, China

**Keywords:** brachytherapy, cervical cancer, complete bicorporeal uterus, genital anomalies, applicator

## Abstract

**Purpose:**

The purpose of this study was to describe an approach to cervical brachytherapy for a patient with a complete bicorporeal uterus and locally advanced cervical cancer (LACC).

**Materials and methods:**

The patient was a 53-year-old woman with a complete bicorporeal uterus, diagnosed with stage IIB cervical squamous cell carcinoma due to contact bleeding. The patient underwent concurrent chemoradiotherapy (CCRT), external beam pelvic radiotherapy with 45 Gy/25 fractions, and weekly cisplatin (40 mg/m^2^). Brachytherapy was administered following the completion of external beam radiotherapy.

**Results:**

The brachytherapy, which was CT (Computed Tomography)-guided using two CT-compatible tandems and two CT-compatible ovoids, delivered a prescription dose of HRCTV D90 was 6 Gy*5F, which achieved satisfactory dose coverage. The patient’s final HRCTV D90 EQD2_10_ was 84.9 Gy, and IRCTV D90 EQD2_10_ was 63.5 Gy. Rectum D2cc EQD2_3_ was 66.03 Gy, bladder D2cc EQD2_3_ was 75.57 Gy, sigmoid D2cc EQD2_3_ was 63.93 Gy, and intestine D2cc EQD2_3_ was 65.86 Gy. Follow-up at 1 year was CR.

**Conclusions:**

For patients with cervical cancer and a complete bicorporeal uterus, using double tandems combined with double ovoids is a feasible treatment method to ensure adequate dose coverage without causing additional damage. This method is also applicable to patients with endometrial cancer.

## Introduction

Cervical cancer is a significant worldwide health issue, with over half a million women diagnosed each year. In high-income countries, the frequency has significantly reduced due to the widespread introduction of cervical screening programs (1). However, many women still die from cervical cancer, particularly in low- and middle-income countries, making it the fourth leading cause of cancer-related death in women worldwide (2). For locally advanced cervical cancer (LACC), concurrent chemoradiotherapy (CCRT) plus brachytherapy is the standard treatment (3, 4). Compared to 2D (two-dimensional) brachytherapy, 3D (three-dimensional) brachytherapy has resulted in an improved tumor control rate and a reduction of radiotherapy’s adverse effects (5, 6). Approximately 4%–7% of females are born with malformations in their reproductive tract, and malformations combined with cervical cancer are rarer. There is no agreed-upon clinical treatment guideline or consensus due to differences in anatomical structure, making brachytherapy challenging. Our patient with a complete bicorporeal uterus and cervical cancer received radical concurrent chemoradiation followed by CT-guided 3D brachytherapy.

## Materials and methods

The 53-year-old female patient experienced a natural birth of twins in 1994 and underwent ovarian cyst removal in 2002, during which she was found to have a complete bicorporeal uterus. Menopause occurred at age 51 years and, in April 2022, she developed contact bleeding. In September 2022, she tested positive for human papillomavirus (HPV) 16 infection and was confirmed by pathological biopsy to have invasive squamous cell carcinoma. Probably because the tumor was small, her squamous cell carcinoma-associated antigen (SCC) test results were normal.

### Staging

**Figure 1:**
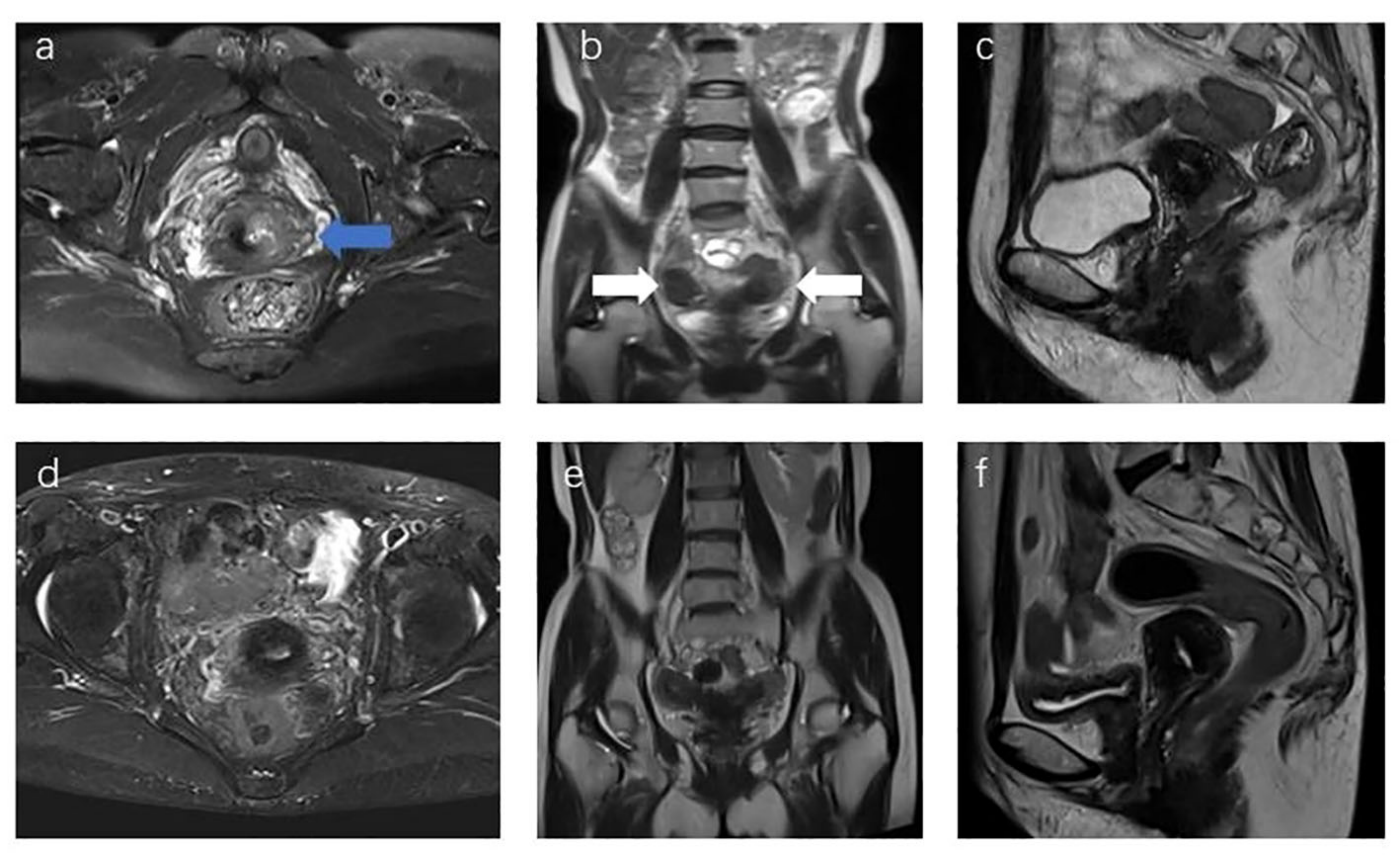
**(A)** MR Sagittal image of the patient before treatment, with the blue arrow showing cervical lesions; **(B)** coronal T2 images of the patient before treatment, with white arrows showing bilateral uterine bodies; **(C)** sagittal image of the patient before treatment. **(D–F)** Re-examination images of the patient 1 year after the end of treatment showed that the lesion was not visible, and the efficacy evaluation was CR.

The patient underwent gynecological and colposcopic examinations, which revealed a mass about 3 cm in diameter on the surface of the cervix. The lesion involved the vagina’s fornix and one-third of the posterior wall, and the left parametrial lesion did not extend to the pelvic wall. Due to financial constraints, the patient did not undergo a Positron Emission Tomography–Computed Tomography (PET-CT) examination. Pelvic magnetic resonance imaging (MRI) showed a cervical tumor measuring 2.7 cm × 2.3 cm × 1.7 cm, which had invaded the left parametrial and vaginal fornix (**Figure 1**). No metastasis was detected in the lymph nodes or other areas. Examination results established that the patient was at cervical squamous cell carcinoma stage IIB (FIGO2018). To conform to standard cervical cancer treatment protocols, the patient received simultaneous chemoradiotherapy followed by brachytherapy.

### Treatment

#### CCRT

The clinical target volume (CTV) of external radiation therapy included the common iliac, internal iliac, external iliac, obturator, anterior sacral lymphatic drainage area, uterus, bilateral parametrial, and upper 2/3 vaginal tissues. The planning target volume (PTV) was derived using a 5-mm 3D external expansion of the CTV, and a prescription dose of 95% PTV DT 45 Gy/1.8 Gy/25F was administered via the infinity linear accelerator with 6 MeV x-ray and VMAT technology. Simultaneously, cisplatin (40 mg/m^2^, 60 mg per session) was administered 5 times per week.

#### Brachytherapy

**Figure 2:**
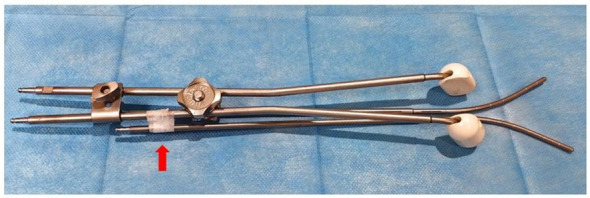
Schematic diagram of applicators, in which one tandem is fixed with a connecting assembly, the upper part of the other tandem is fixed with gauze inside the vagina and the lower part with tape (red arrow indicated).

**Figure 3:**
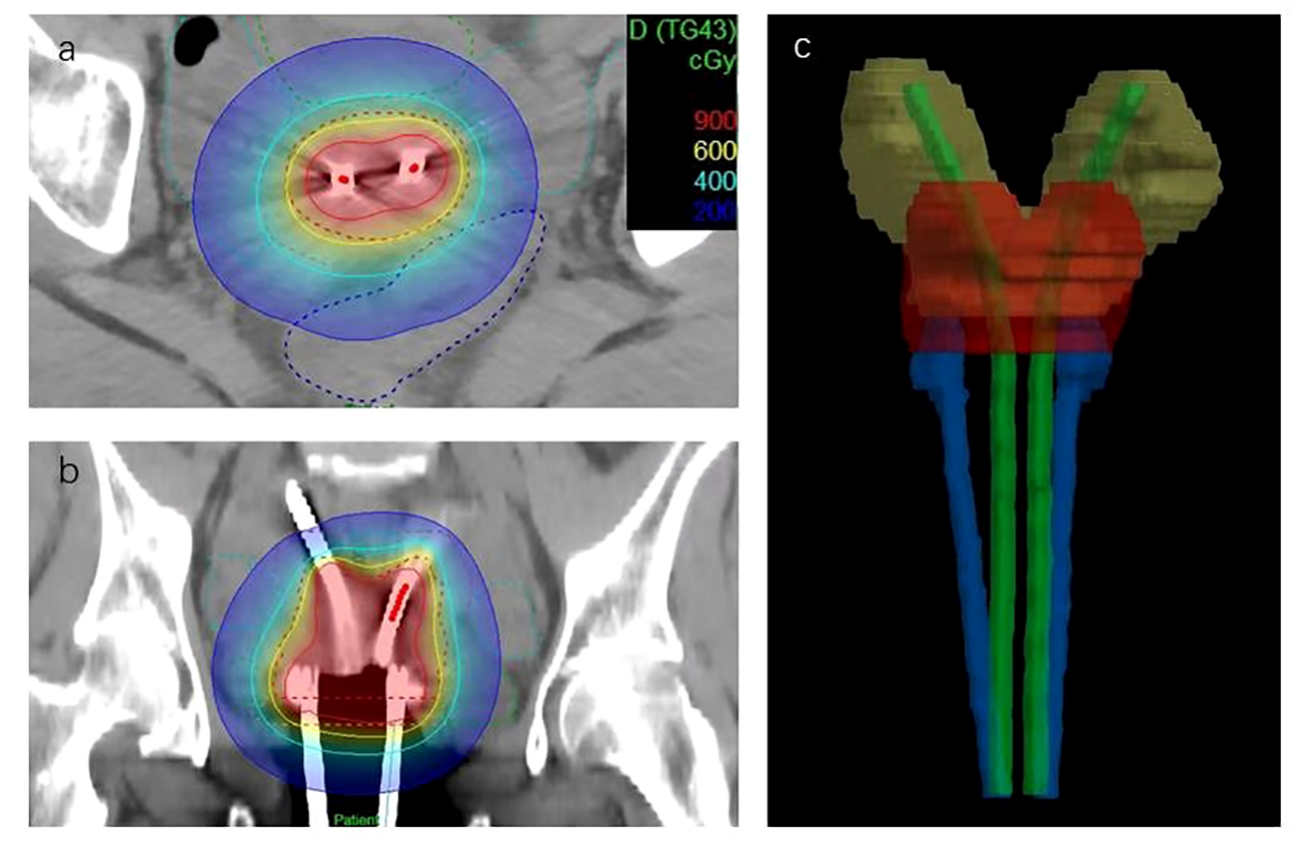
Brachytherapy treatment plan. **(A, B)** Dose distribution of patients in coronal and sagittal positions. The red dashed line in the figure is HRCTV. **(C)** The reconstructed image of the applicators, HRCTV and uterus.

The prescription dose for the high-risk CTV (HRCTV) D90 was 6Gy*5F, and intersegmental CT-guided 3D brachytherapy was adopted. Target delineation referred to the ICRU-89 report and the IBS-GEC ESTRO-ABS CT-Guided Brachytherapy target delineation (7). HRCTV includes the entire cervix and vaginal fornix, which is expanded outward from the basis of HRCTV to form the Intermediate-Risk CTV(IRCTV). The following dosage limits need to be maintained: HRCTV D90 ≥ 80 Gy, IRCTV D90 ≥ 60 Gy, rectum D2cc < 75 Gy, bladder D2cc < 90 Gy, sigmoid D2cc < 75 Gy. Brachytherapy applicator was selected with CT-compatible tandems and ovoids (**Figure 2**). One of the treatment plans and applicators reconstruction images is shown in **Figure 3**.

## Results

The total treatment time was 49 days. The total therapeutic dose for the patient’s brachytherapy target area and organs at risk (OARs) is detailed in **Table 1**. After dose conversion and superposition of the patient’s external irradiation dose, the patient’s final HRCTV D90 EQD2_10_ was 84.9 Gy, and IRCTV D90 EQD2_10_ was 63.5 Gy. Rectum D2cc EQD2_3_ was 66.03 Gy, bladder D2cc EQD2_3_ was 75.57 Gy, sigmoid D2cc EQD2_3_ was 63.93 Gy, and intestine D2cc EQD2_3_ was 65.86 Gy. One year following the conclusion of the patient’s treatment, their MRI review showed complete remission (**Figure 1**), with no observable adverse reactions.

**Table 1:** Brachytherapy (BT) and external beam radiotherapy(EBRT) dose summary.

Treatment fractionation	HRCTV D90	IRCTV D90	Bladder D2cc	Rectum D2cc	Sigmoid D2cc	Intestine D2cc
1	6.04 Gy	3.30 Gy	4.05 Gy	3.31 Gy	2.70 Gy	3.23 Gy
2	6.01 Gy	3.03 Gy	3.75 Gy	4.07 Gy	2.45 Gy	3.25 Gy
3	6.08 Gy	3.61 Gy	4.51 Gy	2.90 Gy	3.73 Gy	3.33 Gy
4	6.13 Gy	3.66 Gy	4.65 Gy	3.54 Gy	3.80 Gy	3.75 Gy
5	6.09 Gy	3.57 Gy	4.89 Gy	3.65 Gy	3.62 Gy	3.86 Gy
BT total dose(EQD2)	40.65 Gy	19.25 Gy	32.37 Gy	22.83 Gy	20.73 Gy	22.66 Gy
EBRT+BT(EQD2)	84.9 Gy	63.5 Gy	75.57 Gy	66.03 Gy	63.93 Gy	65.86 Gy
Target dose(EQD2)	≥80 Gy	≥60 Gy	≤90 Gy	≤75 Gy	≤75 Gy	≤75 Gy

Dose conversion and superposition were performed for the patients with brachytherapy, where HRCTV and IRCTV α/β = 10, and α/β = 3 for organs at risk. The calculated EBRT dose EQD2_10_ = 44.25 Gy, EQD2_3_ = 43.2 Gy.

## Discussion

CCRT combined with brachytherapy is the standard treatment for LACC. Intracavitary brachytherapy has less impact on OARs due to its high-dose gradient and is therefore essential for cervical cancer treatment. The key to successful brachytherapy is selecting a suitable applicator to achieve satisfactory dose distribution. Conventional applicators are designed for normal anatomical structures, and there are no individualized applicators for patients with reproductive tract malformations.

Female reproductive tract malformation refers to a variety of structural abnormalities in the fallopian tubes, uterine body, cervix, and vagina caused by abnormalities in different nodes during the development, fusion, and absorption of Mullerian canal. These abnormalities may consist of a solitary local anomaly, such as the mediastinum of the uterus, or a combination of abnormalities across multiple sites. The main categories of female reproductive tract malformations include the American Fertility Society Classification of Mullerian Tube Abnormalities (1988), the European Society of Human Reproduction and Embryology and European Society of Gynecological Endoscopy (ESHRE/ESGE) Classification of Congenital Abnormalities of the female reproductive tract (2013), the Vagino-Cervico-Uterine-Adnex-Associated Malformations Classification (2005), and the Embryological and Clinical Classification of Female genito-urinary system Malformations (2011 update). In this case, the ESHRE/ESGE classification was used, and the patient was identified as having a U3/b complete double uterus (8).

There is no standard radiotherapy protocol for cervical cancer patients with genital tract malformations. The target area of external irradiation includes all uterine bodies and the cervix, with other target areas and prescription doses remaining the same as those for conventional patients. Due to anatomical abnormalities, brachytherapy is challenging, resulting in variations in treatment plans. We reviewed relevant literature and found several similar case reports. H W LOO et al., aiming at a patient with double uterus and double cervix, adopted a brachytherapy program of bilateral alternating uterine cavity (9). Guler Yavas et al. applied a special vaginal mold to a patient with stage IIIA cervical adenocarcinoma with a double uterus and double cervix and performed pulsed brachytherapy with intertissue insertion (10). Platta et al. treated a case of stage IIB cervical adenocarcinoma of the mediastinal uterus with a Rotte-Y tandem and two domes (11). ChengzhiLei et al. used a vaginal mold for a patient with double uterine cervical clear cell carcinoma of stage IIB and administered two separate doses of 7 Gy on the left and right sides using a uterine tube plus insertion needle (12). C D LEE et al. performed 2D brachytherapy with bilateral uterine ducts on a patient with cervical squamous cell carcinoma with a double cervix and double uterus (13). Denise Fabian et al. treated a patient with a bicornis uterus using a ring applicator combined with an insertion needle (14). Abel Cordoba et al. applied a 3D-printed vaginal model to a cervical cancer patient with HWW (Herlin-Werner-Wunderlich) syndrome for intertissue implantation brachytherapy (15).

For this patient, the disease was at stage IIB, and the residual lesion was found in the cervix after external irradiation treatment. For regular patients, single tandems and double ovoids are viable options. Single tandem and Rotte-Y tandem cannot provide sufficient dose coverage due to the considerable spacing between bilateral uterine cavities. After a thorough discussion, we employed a novel treatment approach using common applicators, two CT-compatible tandems, and two CT-compatible ovoids for this patient. Given the large bilateral uterine distance, 30° tandems were selected. During treatment, one tandem was attached to the ovoids using connecting components, while the other was secured with tape and additional components. After filling the vagina with gauze, each applicator was securely fixed. The prescription dose line fully covered the target area and bilateral lower uterine segments, achieving satisfactory dose coverage with reliable consistency between sessions. After five sessions, the cumulative dose met the requirements for HRCTV D90 and IRCTV D90, and the OARs remained within acceptable limits. It should be noted that our therapy machine, the Flexitron-HDR, has a total of 10 channels. Only the fifth channel can connect to the tandem. Thus, our treatment plan had to be divided into two parts: in the first part, we plan to use channels 1, 3, and 5 to connect two ovoids and one tandem, and use channel 5 to connect another tandem after completing the first part of the treatment.

According to the ESHRE/ESGE classification method, the Rotte-Y tandem is suitable for cases of mediastinal uterus, conventional treatments are applicable for cases of uniangular uterus, and our method is recommended for patients with a double uterus. If the remaining lesion is too extensive or there is an abnormality in the uterus, interstitial treatment may be combined (**Table 2**). Additionally, the utilization of 3D-printing technology can be considered to enhance the distribution of the emitter and improve dose coverage. This treatment offers several benefits: first, conventional applicators were selected, ensuring replicable evaluation and measurement, which can be carried out at treatment institutions of all tiers. Second, it does not increase patient injury compared to intertissue implantation. Finally, it provides a more flexible angle compared to the Rotte-Y tandem, allowing for satisfactory coverage of the uterine body with different tandem combinations. This method of treatment has some limitations. Patients with cervical stenosis or adhesion need dilation, as the double uterine duct diameter is larger and requires a certain cervical duct width to be treated. Additionally, the treatment plan needs to be divided into two parts due to the uniqueness of the uterine tandem connection tube. This theoretically will not cause a deviation in the therapeutic dose, but it increases the difficulty of treatment.

**Table 2:** Comparison of different treatment modalities.

Treatment	Advantage	Disadvantage	Applicable patient
DT+DO	Noninvasive; Tandems can be rotatable; Simple applicator.	Need to dilate the cervix; Treatment plan need to be phased; Lack of fixed structure	Endometrial cancer; Cervical cancer with bicor-poreal uterus or mediastinal uterus; Patients with small tumors;
Y+DO	Noninvasive; Simple to operate	Requires a unique Y-tandem; Tandems can’t spin;	
ISBT	Flexible treatment plan; 3D printable	Invasive, pain, bleeding; High cost; Difficult to operate	Patients with large tumors; Standard applicators are not suitable.

DT, double tandem; DO, double ovoid; Y, Y-tandem; ISBT: interstitial brachytherapy; 3D: three-dimensional.

For people with endometrial cancer that cannot be operated on, radiation treatment increases the chance of survival (16). For the brachytherapy of endometrial cancer, the special Rotte-Y tandem is often selected (17). The technique we employed is suitable for administering brachytherapy to patients with endometrial cancer, particularly in institutions that do not provide the Rotte-Y tandem. We treated a case of inoperable endometrial cancer, and the combination of two 15° tandems and double ovoids achieved very satisfactory dose coverage without increasing the patient’s risk of injury or treatment difficulty.

The implementation of 3D brachytherapy technology and the availability of various applicator types provide more treatment options for patients with reproductive tract malformations. However, due to the diversity of deformities, the range of applicators currently in use is limited. Therefore, treating such patients often necessitates the use of multiple applicators in combination to achieve satisfactory therapeutic outcomes.

## Conclusions

For cervical cancer patients with a complete bicorporeal uterus, the use of double tandems combined with double ovoids is a feasible treatment alternative that achieves adequate dose coverage without causing additional harm. This therapeutic approach may also be applicable to individuals diagnosed with endometrial cancer.

## Data availability statement

The original contributions presented in the study are included in the article/supplementary material. Further inquiries can be directed to the corresponding author.

## Ethics statement

Written informed consent was obtained from the individual(s) for the publication of any potentially identifiable images or data included in this article.

## Author contributions

LD: Writing – original draft, Writing – review & editing. ZZ: Writing – original draft. YC: Data curation, Writing – original draft. ZW: Data curation, Writing – original draft. BZ: Data curation, Writing – original draft. GM: Data curation, Writing – original draft. WY: Writing – review & editing.
